# Diagnostic Work-Up of a Giant Calcified Intracranial Aneurysm: Comparing 4D-CTA and Cerebral Angiogram Findings

**DOI:** 10.7759/cureus.1367

**Published:** 2017-06-19

**Authors:** Layth Dahbour, Tarek R Mansour, Ahmed Alnemari, Mark Buehler, Daniel Gaudin

**Affiliations:** 1 Department of Surgery, Division of Neurosurgery, The University of Toledo Medical Center; 2 Department of Radiology, The University of Toledo Medical Center

**Keywords:** giant calcified aneurysm, cerebral angiography, dynamic ct angiography, mca aneurysm

## Abstract

The risks associated with unruptured intracranial aneurysms can be neurologically debilitating and even fatal. Evaluation of these aneurysms is critical for determining what type of intervention is warranted, if at all. Cerebral angiography has long been the gold standard in the evaluation of intracranial aneurysms. However, this diagnostic modality is accompanied by several risks that are made clear to the patient before they consent to the procedure. These risks include the possibility of stroke, groin hematomas, contrast-induced anaphylaxis, contrast nephropathy, and catheter-associated infections. Dynamic CT angiography (4D-CTA) has been studied as an assessment tool for cerebral vasculopathies such as stroke, arteriovenous malformations, and aneurysms. It has been shown that 4D-CTA has the advantage of being less invasive and has a shorter examination time than cerebral angiography. In this article, we present a rare case of a giant calcified aneurysm and compare the findings of a cerebral angiogram and a 4D-CTA study.

## Introduction

Approximately two to five percent of the general population have an intracranial aneurysm [1]. Rupture resulting in subarachnoid hemorrhage is the most severe complication among these patients. Moreover, aneurysmal calcification has been shown to predict poor post-surgical outcomes [2]. Therefore, assessing the use of different imaging modalities is warranted in the management of these patients. Cerebral angiography has been the gold standard in the evaluation of aneurysms; however, this invasive procedure is accompanied by many disadvantages [3].

Dynamic computed tomography angiography (4D-CTA) has been studied as an assessment tool for cerebral vasculopathies such as stroke, arteriovenous malformations, and aneurysms. The 4D-CTA modality combines the noninvasive nature of conventional computed tomography angiography (CTA) with the dynamic capability of conventional angiography to characterize neuro-vascular structures and illustrate their flow dynamics through continuous volumetric computed tomography (CT) acquisition [4-5]. Recently, it has been reported that 4D-CTA has the potential to appreciate aneurysmal wall motion and pulsation which may better predict rupture risk [5-6]. Furthermore, 4D-CTA has the advantage of being less invasive and having a shorter examination time than cerebral angiography. We present a rare case of a giant calcified aneurysm and compare the findings of a cerebral angiogram and a 4D-CTA study.

## Case presentation

A 73-year-old male presented to an outside hospital complaining of dizziness and vertigo with occasional jerking of his right lower extremity while walking. Past medical history includes diabetes mellitus, hyperlipidemia, congestive heart failure, gastroesophageal reflux disease, and valvular heart disease. The patient has no history of drug or alcohol use, but was previously a smoker. The patient was later transferred to our university hospital and was admitted to the neurosurgery service for further evaluation of his symptoms. Upon presentation, the patient’s physical and neurological exam was unremarkable and the patient had a Glasgow Coma Score of 15.

**Figure 1:**
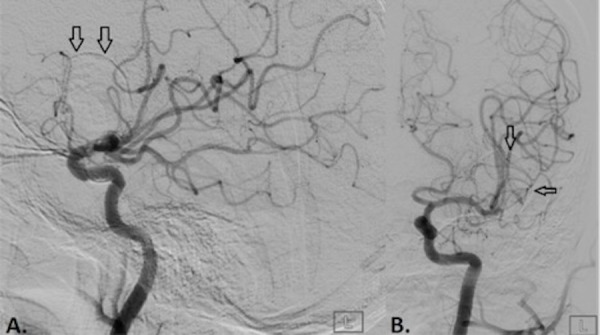
Conventional digital subtraction angiographic images from the left internal carotid artery in lateral (A) and frontal (B) projection. The images show the faint outline of a partially calcified giant left middle cerebral artery bifurcation aneurysm (black arrows). The aneurysm did not demonstrate contrast filling indicating that it was completely thrombosed.

**Figure 2:**
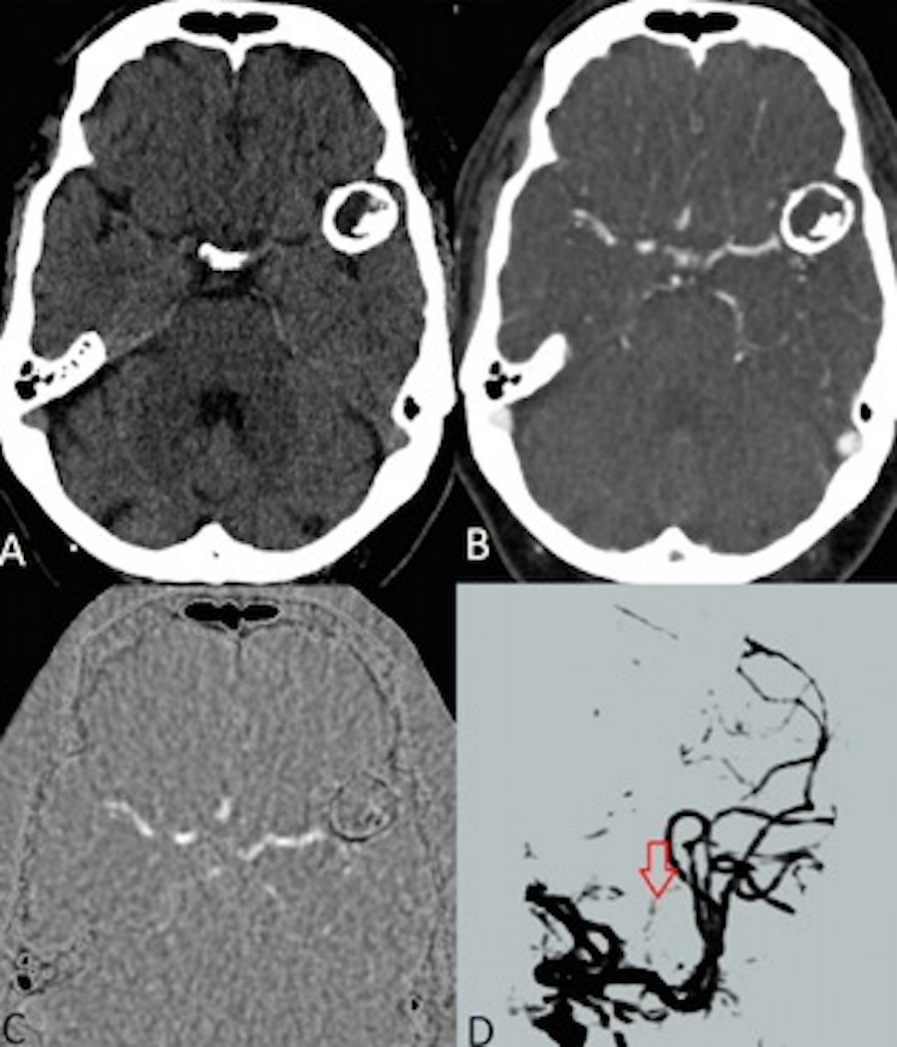
Images from the 4D-digital subtraction CT angiogram. The non-contrast head CT (A) shows a partially calcified aneurysm with a non-calcified component along the anterior/medial wall. An image from the late arterial/early venous phase of the contrast enhanced portion of the study (B) shows no evidence of contrast opacification within the non-calcified component. The two studies are digitally subtracted (C) to further demonstrate the lack of contrast opacification. The data from the arterial, parenchymal, and venous phases are compiled to make a digitally subtracted three dimensional, temporally resolved CT angiogram that looks similar to the conventional angiographic images (D). No contrast fills the aneurysm lumen. The calcified, incompletely subtracted wall (red arrow) is all that can be seen of the aneurysm.

The patient was transferred to our hospital with a non-contrast head CT study done at the initial outside facility. The image showed a giant calcified left middle cerebral artery (MCA) mass upon admission to our academic medical center. A diagnostic cerebral angiogram and 4D-CTA study were ordered for further work-up and evaluation of aneurysmal blood flow. The angiogram showed no intracranial aneurysm opacification (Figure 1). Analysis of the 4D-CTA study showed a 2.7 x 2.4 x 3.0 cm left MCA calcified aneurysm with no internal enhancement on contrast images (Figure 2).

## Discussion

The risks associated with a giant unruptured aneurysm warrant a thorough work-up that elucidates several characteristics such as size, calcification, blood flow, and thrombosis. These are used to help assess rupture risk and the postoperative outcome. Neurosurgeons, interventional neurologists, and neuroradiologists rely heavily on imaging modalities to determine whether a particular intervention such as surgical clipping or endovascular coiling should be indicated for a patient. In a single-institution study comparing patients with unruptured intracranial aneurysms who underwent either coiling or clipping, Bhatia et al. showed that calcification was a sole marker of adverse clinical outcomes in the case of an unruptured aneurysm, whereas size alone was not. Moreover, poor postoperative outcomes were more likely by about eight-fold in the presence of aneurysmal calcification. They concluded that calcification of the neck may uniquely complicate the surgical procedure of clip placement requiring consideration of other methods such as the use of hypothermic circulatory arrest which is not typically desirable [2,7].

In this case, the diagnostic work-up of the patient allowed for comparing cerebral angiography and 4D-CTA imaging, techniques that have been widely used in the setting of diagnosing intracranial aneurysms. Both imaging modalities were used to assess rupture risk, and it was reassuring to discover that both yielded similar findings and ultimately showed no aneurysmal blood flow likely due to a completely thrombosed lumen. The angiogram showed no contrast filling of the calcified aneurysm, while the 4D-CTA showed no contrast opacification within the non-calcified component of the aneurysm.

The perioperative risks of giant calcified intracranial aneurysms necessitate advanced, yet minimally invasive diagnostic testing that can precisely guide care providers in the treatment of these patients. Recently, Vanrossomme et al. proposed 4D-CTA as an imaging modality that could assess aneurysmal wall motion as a potential predictor of rupture risk [5]. This new approach could better utilize the modality and allow it to provide new measures that can better predict rupture risk and therefore accurately guide providers in comparing the risks and benefits associated with a specific intervention. CTA also possesses several advantages such as a shorter examination duration, increased scanner availability, and improved spatial resolution [4]. Although it possesses some disadvantages such as increased radiation exposure and the need for iodine contrast, it bypasses the unique risks of the alternative modality, in this case, cerebral angiography.

## Conclusions

In the presence of a giant calcified aneurysm, 4D-CTA showed similar findings to that of a cerebral angiogram. Given its increasing accessibility, potential to provide new rupture risk measures, and advantages over cerebral angiography, 4D-CTA is emerging as a favorable imaging modality in the diagnosis and follow-up of patients with giant calcified intracranial aneurysms.
